# Cold Plasma Inactivation of Bacterial Biofilms and Reduction of Quorum Sensing Regulated Virulence Factors

**DOI:** 10.1371/journal.pone.0138209

**Published:** 2015-09-21

**Authors:** Dana Ziuzina, Daniela Boehm, Sonal Patil, P. J. Cullen, Paula Bourke

**Affiliations:** 1 Plasma Research Group, School of Food Science and Environmental Health, Dublin Institute of Technology, Dublin 1, Ireland; 2 School of Chemical Engineering, University of New South Wales, Sydney, Australia; Ghent University, BELGIUM

## Abstract

The main objectives of this work were to investigate the effect of atmospheric cold plasma (ACP) against a range of microbial biofilms commonly implicated in foodborne and healthcare associated human infections and against *P*. *aeruginosa* quorum sensing (QS)-regulated virulence factors, such as pyocyanin, elastase (Las B) and biofilm formation capacity post-ACP treatment. The effect of processing factors, namely treatment time and mode of plasma exposure on antimicrobial activity of ACP were also examined. Antibiofilm activity was assessed for *E*. *coli*, *L*. *monocytogenes* and *S*. *aureus* in terms of reduction of culturability and retention of metabolic activity using colony count and XTT assays, respectively. All samples were treated ‘inpack’ using sealed polypropylene containers with a high voltage dielectric barrier discharge ACP generated at 80 kV for 0, 60, 120 and 300 s and a post treatment storage time of 24 h. According to colony counts, ACP treatment for 60 s reduced populations of *E*. *coli* to undetectable levels, whereas 300 s was necessary to significantly reduce populations of *L*. *monocytogenes* and *S*. *aureus* biofilms. The results obtained from XTT assay indicated possible induction of viable but non culturable state of bacteria. With respect to *P*. *aeruginosa* QS-related virulence factors, the production of pyocyanin was significantly inhibited after short treatment times, but reduction of elastase was notable only after 300 s and no reduction in actual biofilm formation was achieved post-ACP treatment. Importantly, reduction of virulence factors was associated with reduction of the cytotoxic effects of the bacterial supernatant on CHO-K1 cells, regardless of mode and duration of treatment. The results of this study point to ACP technology as an effective strategy for inactivation of established biofilms and may play an important role in attenuation of virulence of pathogenic bacteria. Further investigation is warranted to propose direct evidence for the inhibition of QS and mechanisms by which this may occur.

## Introduction

Bacterial biofilms represent a significant source of human infections, which may be acquired through interaction with a wide range of everyday or clinical environments including the consumption of contaminated foods, via hospital environment, medical equipment or devices [1–2]. In the United States approximately 80% of persistent bacterial infections are associated with biofilms [3]. Biofilms are defined as complex microbial communities enclosed in hydrated extracellular polymeric substances (EPS), which comprise polysaccharides, proteins, phospholipids, teichoic and nucleic acids [4]. Due to their heterogeneous nature, bacterial biofilms are characterized with an enhanced resistance by comparison with their planktonic counterparts to most environmental stresses including nutrient starvation, oxidative stress, antibiotic exposure and other conditions detrimental to bacterial growth [5–6]. In turn, these stresses can act as determinants for biofilm formation; indeed the transition from planktonic to biofilm form can be considered as a stress response mechanism [7–8]. One of the regulatory mechanisms that bacteria employ to respond to external environmental stresses through the expression of a large number of genes is quorum sensing (QS), widely defined as a population-controlled bacterial communication process [9]. QS regulates numerous important cell functions in both Gram-positive and Gram-negative bacteria, including metabolism, protein synthesis, expression of virulence factors, antibiotic resistance, biofilm formation, biofilm maintenance and dispersal, and entry to stationary phase, therefore, QS is a highly attractive target in the search for alternative antimicrobial agents [7, 9–10]. *Pseudomonas aeruginosa* is an important clinical pathogen that is extensively studied as a model organism with respect to QS as well as biofilm formation. *P*. *aeruginosa* possesses two individual but interconnected acyl-homoserine lactone (AHL) based QS signalling systems, *las* and *rhl* [10]. The *las* system regulates the production of elastases (Las B), protease (Las A), exotoxins and development of biofilms, whereas *rhl* induces the production of elastase, proteases, rhamnolipid, pyocyanin and siderophores [11–12]. There is a third QS signalling molecule in *P*. *aeruginosa* termed as *Pseudomonas* quinolone signal (PQS), which is also involved in the pyocyanin synthesis. Pyocyanin, which is a redox-active, blue pigment phenazine, toxic to prokaryotic and eukaryotic cells, is one of the most important QS-controlled virulence factors produced by *P*. *aeruginosa* [13]. By interfering with a wide spectrum of cellular functions in host cells such as electron transport, cellular respiration, energy metabolism, gene expression, ciliary function, epidermal cell growth and innate immune mechanisms, pyocyanin contributes to chronic respiratory infections and sepsis [14–15]. Another highly toxic virulence factor secreted by *P*. *aeruginosa* is the metalloprotease elastase, also known as Las B, which causes tissue damage and invasion, by degradation of elastin, fibrin and collagen, inactivation of the immune system components, and acts intracellularly to initiate bacterial biofilm growth [16–17]. The key role of these QS-controlled virulence factors in the pathogenesis of *P*. *aeruginosa* indicates a need to examine and expand antimicrobial strategies targeting not only structural components of biofilms as well as inactivation of bacterial cells within biofilm complexes but also specifically the inhibition of virulence factors so that an improved outcome of microbial biofilm inactivation and control of microbial infection are achieved.

Atmospheric cold plasma (ACP) is a relatively novel approach increasingly studied against a wide range of microbial pathogens either in planktonic or biofilm form. ACP is often referred to as the fourth state of matter, which under the constant supply of energy to a gas results in the generation of photons, electrons, positively and negatively charged ions, atoms, free radicals and excited or non-excited molecules [18]. Although the exact mechanism of ACP bactericidal action is yet to be elucidated, it is known that atmospheric air ACP is a source of multiple chemically reactive species, including reactive oxygen (ROS) and reactive nitrogen species (RNS), with a high bactericidal potency. Among the ROS, ozone, atomic oxygen, singlet oxygen, superoxide, peroxide, and hydroxyl radicals, independently or in synergy, are expected to play a role in the bacterial inactivation process [19–20]. Our previous studies demonstrated that ACP in conjunction with in-package treatment was very effective for reducing high concentrations of planktonic bacterial population to undetectable levels within short treatment times [21]. However, a reduced antimicrobial effect of ACP treatments against bacteria growing in a biofilm form has been reported [22–24]. Due to the challenge presented by the persistence of biofilms and lack of techniques to overcome these challenges, an effective control of bacterial biofilms is becoming crucial and should play an important role in the design of disinfection strategies. Moreover, because QS may be involved in the formation of biofilms [25], it is of fundamental research interest to investigate whether ACP technology can specifically interrupt or inhibit bacterial QS systems through interference with virulence factor production. Therefore, the main objectives of this study were to investigate the potential of ACP for elimination of challenge monoculture biofilms and to examine inhibition potential against bacterial virulence factors. Surviving bacterial populations in established biofilms of *E*. *coli*, *L*. *monocytogenes* and *S*. *aureus* were estimated by colony count and XTT assays for determination of cell viability and metabolic activity, respectively. Scanning electron microscopy (SEM) analysis was conducted to observe any morphological changes of biofilms caused by ACP treatment. Inhibition of QS was studied only for *P*. *aeruginosa* which was selected as the model organism with respect to QS by measuring concentrations of pyocyanin (*rhl*), elastase production (*las*) and biofilm formation capacity post ACP treatment. Finally, in order to examine if ACP-based reduction of virulence factors could further influence the toxicity of *P*. *aeruginosa*, a cytotoxicity assay using CHO-K1 cell line was conducted. The effects of the processing factors of treatment time and mode of plasma exposure on antimicrobial activity of ACP were examined.

## Materials and Methods

### Bacterial strains and growth conditions

Bacterial strains utilised in this study were *Escherichia coli* NCTC 12900, obtained from National Collection of type cultures of the Health Protection Agency (HPA, UK), *Staphylococcus aureus* NCTC 1803, *Listeria monocytogenes* NCTC 11994 and *Pseudomonas aeruginosa* ATCC 27853, which were obtained from the microbiology stock culture of the School of Food Science and Environmental Health of the Dublin Institute of Technology. A single isolated colony of each culture was inoculated in tryptic soy broth without glucose (TSB, ScharlauChemie, Spain). For biofilm studies, *E*. *coli*, *L*. *monocytogenes* and *S*. *aureus* cultures were incubated for 18 h at 37°C, which were further diluted in TSB to a cell density of 1.0 × 10^7^ CFU/ml and used as inoculum for biofilm formation. *P*. *aeruginosa* was inoculated in TSB and incubated for 24 h at 37°C and further used in inhibition of QS-controlled virulence factors, i.e. pyocyanin, elastase (Las B) and biofilm formation experiments. Separate experiments to determine pyocyanin levels were conducted using 24 h grown *P*. *aeruginosa* culture cell free supernatant.

### Cultivation of CHO-K1 cells

Chinese hamster ovary cells (CHO-K1) were obtained from biological stock culture of the School of Food Science and Environmental Health of the Dublin Institute of Technology. The cells were grown in Dulbecco's Modified Eagle's Medium/Ham's F-12 Nutrient Mixture (DMEM/F12, Sigma-Aldrich Co., Ireland) containing 2 mM L-glutamine and 10% (v/v) foetal bovine serum (FBS) at 37°C and 5% carbon dioxide (CO_2_). Confluent cell layers were washed with phosphate buffered saline (PBS, Sigma-Aldrich Co., Ireland) and enzymatically detached using trypsin/EDTA.

### Biofilm formation

A 96 well microtiter plate method was used for biofilm formation. Briefly, either *E*. *coli*, *L*. *monocytogenes* or *S*. *aureus* TSB cell suspension (200 μl) was added into the wells (8 wells for each replicate) and incubated at 37°C for 48 h, replacing the supernatant with fresh TSB after the first 24 h of incubation. For scanning electron microscopy (SEM) analysis, *E*. *coli* biofilms were produced using 6 well inserts containing track-etched polyethylene terephthalate (PET) membranes with 0.4 μm pore size (Becton Dickinson Labware, USA). The inserts were placed inside the wells of the 6 well culture plate, filled with 2 ml of TSB bacterial suspension (1.0 × 10^7^ CFUml^-1^) and incubated for 48 h at 37°C, changing the supernatant after 24 h of incubation. After 48 h of incubation, the supernatant from either microplate wells or inserts was removed and substrates were rinsed three times with sterile PBS, leaving only bacterial biofilms for further investigation. Negative controls were obtained by using uninoculated TSB. Prior to each experiment, the microtiter plates and 6 well inserts containing biofilms were air dried for approximately 60 min.

### Experimental design

The ACP system utilised was a DBD system previously described in [21], with a maximum high voltage output of 120 kV at 50 Hz and 22 mm distance between the two aluminium electrodes. The distance was equal to the height of the polypropylene container (310 x 230 x 22 mm), which was used as both a sample holder and as a dielectric barrier. Plasma was generated in air at 80 kV_RMS_.

**Fig 1:**
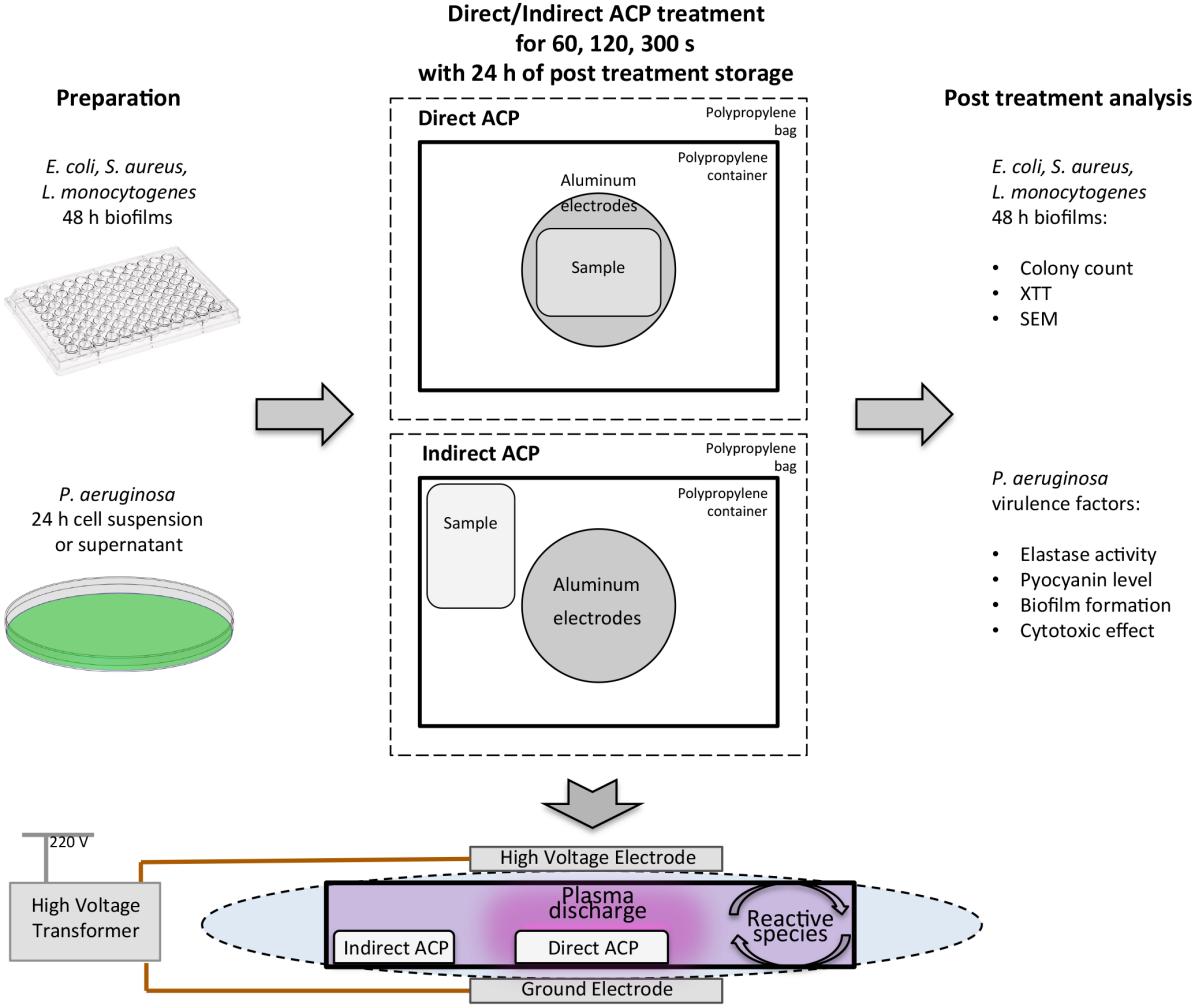
Experimental set up. Either a microtiter plate containing bacterial biofilms or a petri dish containing *P*. *aeruginosa* cells TSB suspension or cell-free supernatant were placed inside the plastic container between the electrodes for direct ACP treatment. For indirect ACP treatment, a separate container was used and samples were placed outside the plasma discharge.

Effects of both direct and indirect ACP treatment were evaluated (Fig 1). Either a microtiter plate or PET membrane insert containing bacterial biofilms or a petri dish containing *P*. *aeruginosa* cells TSB suspension or *P*. *aeruginosa* cell-free supernatant (10 ml) were placed in the centre of the plastic container directly between the electrodes within the plasma discharge for direct ACP treatment and were thus exposed to all generated reactive species, such as charged particles, positive and negative ions, electrons, free radicals, excited and non-excited molecules and atoms, heat and UV photons [26]. The distance between the sample and top electrode for direct treatment was approximately 10 mm. For indirect ACP treatment, a separate container was used and samples were uniformly placed at some distance from the plasma discharge [21] so as to achieve treatment outside the plasma discharge where the antimicrobial effects are mainly attributed to long-lived radicals and recombined products [26]. The distance between the samples and centre of the electrodes for indirect treatment varied from 120 mm to 160 mm due to samples distribution in the microtiter plate or in petri dish. After sample loading, each polypropylene container was sealed with a high barrier polypropylene bag (Cryovac, B2630, USA) and placed between the aluminium electrodes of the transformer. The samples were treated with either direct or indirect ACP for 60, 120 and 300 s. The temperature of the surface of TSB cell suspension and the 96 well plate measured immediately after 300 s of direct/indirect treatment using IR thermometer (Maplin Electronics, S63 5DL, UK) was 21/17°C and 24/18°C, respectively, whereas the temperature measured at the centre of the polypropylene container did not exceed 28°C. All treated samples were subjected to a post treatment storage time of 24 h at room temperature. Control samples were left untreated (0 h control) to estimate initial bacterial concentration, elastolytic activity and pyocyanin levels. To assess possible effects of 24 h of post treatment storage on bacterial viability and enzymatic activity a separate set of untreated samples were stored for 24 h under conditions identical to post treatment storage. All biofilm-based experiments were conducted by using three independently grown cultures and repeated at least twice.

### Biofilm assays

#### Colony count assay

To determine the effect of ACP treatment on the viability of cells within biofilms formed in 96 well microtiter plates, 200 μl of sterile PBS were added into the wells. The plates were then sonicated using a water table sonicator (Bransonic 5510E-MT, USA, Mexico) for 5 min in order to disrupt biofilms. PBS cell suspensions of corresponding culture were pooled together from the wells into sterile eppendorf tubes and serially diluted in maximum recovery diluent (MRD, ScharlauChemie, Spain). Aliquots of appropriate dilutions (100 μl) were surface plated on TSA and incubated at 37°C for 24–48 h. Results were presented as surviving bacterial population in Log_10_ CFUml^−1^ units.

#### XTT assay

To examine the effect of ACP treatment on metabolic activity of bacterial cells in biofilms a 2,3-bis (2-methoxy-4-nitro-5-sulfophenyl) [phenyl-amino)car-bonyl]-2H-tetrazolium hydroxide assay (XTT, 1 mg/ml, Sigma-Aldrich Co., Ireland) was utilised following the procedure previously described by [27]. Briefly, wells containing biofilms and negative controls (TSB without inocula) were filled with sterile PBS (100 μl) and fresh solution of XTT-menadione (100 μl) was added to all wells. Following incubation for 5 h at 37°C in the dark, supernatant (100 μl) from each well was transferred into the wells of a new 96 well microtiter plate and the absorbance was measured at 486 nm on a microplate reader (Synergy HT, Biotek Instruments Inc.). The percentage of surviving bacterial population was calculated as ((A_ACP_-A_C_)/A_0_) X100%, where A_ACP_, A_C_ and A_0_ are the absorbance of ACP treated, negative control and untreated control biofilms, respectively.

#### Scanning electron microscopy (SEM)

*E*. *coli* biofilms; untreated 0 h controls and samples directly treated with ACP for 300 s were selected for SEM analysis. Following fixation for 2 h in ice-cold 2.5% glutaraldehyde in 0.05 M sodium cacodylate buffer (pH 7.4) (SCB), cells were washed with the same buffer and fixed in 1% osmium tetroxide for 2 h at 4°C. After 2 h of fixation, cells were washed with SCB followed by three washes with distilled water. Samples were treated with increasing concentrations of ethanol (30%, 50%, 70%, 80%, 95%, and 99.5%) and dehydrated using 33%, 50%, 66%, and 100% (v/v) hexamethyldisilazane (Sigma Aldrich, Ireland). Samples were sputter-coated with gold particles using an Emitech K575X Sputter Coating Unit resulting in a coating of 10 nm after 30 s. The samples were examined visually using a FEI Quanta 3D FEG Dual Beam SEM (FEI Ltd, Hillsboro, USA) at 5 kV.

### *P*. *aeruginosa* QS-controlled virulence factors assays

#### Pyocyanin assay

Pyocyanin (*rhl*-controlled QS system) present in *P*. *aeruginosa* TSB suspension or supernatant was extracted following the procedure described by [28]. Briefly, either untreated (0 h and 24 h controls) or ACP treated and stored for 24 h post treatment bacterial cell suspensions or culture supernatants, were centrifuged for 10 min at 10,000 rpm. Each supernatant (5 ml) was transferred into a 25 ml tube containing 3 ml of chloroform and the resulting chloroform layer (3 ml) was mixed with 1.5 ml of 0.2 M hydrochloric acid. The solution containing extracted pyocyanin (top layer) was transferred into the wells of a 96 well plate and the absorbance was measured using a microtiter plate reader at 520 nm. An average absorbance value obtained from 10 wells was corrected by subtracting the mean of the absorbance of a blank (uninoculated) TSB. Experiments were repeated at least three times.

#### Elastin-Congo red assay

The effect of ACP treatment on *P*. *aeruginosa* elastolytic activity (Las B), controlled by the *las* QS system, was determined using elastin-Congo red conjugate (Sigma Aldrich, Ireland), as the most specified substrate for elastase, following the procedure described by [29]. Either untreated 0 h and 24 h controls or ACP treated bacterial suspensions were centrifuged for 10 min at 10,000 rpm. Elastin-Congo red (2.5 mg) was suspended in 500 μl of 0.2 M Tris buffer (pH 8.8) in an eppendorf, vortexed, and mixed with 500 μl of *P*. *aeruginosa* supernatant. The resulting solution was incubated at 37°C for 24 h. After incubation, the samples were vortexed and centrifuged at 13,000 rpm for 10 min. The supernatant was dispensed into the wells of the 96 well microtiter plate and the absorbance of released Congo red was measured at 495 nm. An average absorbance, corrected by the mean absorbance obtained from the corresponding mixture incubated in the absence of elastin-Congo red, represents *P*. *aeruginosa* enzymatic elastase activity. Experiments were repeated at least five times.

#### Planktonic cell population density

The possible changes in concentrations of *P*. *aeruginosa* planktonic cell populations caused by ACP treatment were monitored conducting colony count assay of bacterial samples treated with ACP either directly or indirectly for 60, 120, and 300 s and untreated 0 h and 24 h controls. Experiments were repeated at least six times and results were presented as surviving bacterial population in Log_10_ CFUml^−1^ units.

#### Biofilm formation assay

The effect of ACP treatment on *P*. *aeruginosa* biofilm formation was examined using colony count assay. Samples treated with ACP and stored for 24 h or untreated 0 h and 24 h controls of *P*. *aeruginosa* TSB suspensions were dispensed into the wells of 96 well microtiter plates (200 μl). The plates were incubated for 24 h at 37°C. After incubation, the reduction in culturability of *P*. *aeruginosa* biofilms was assessed by colony count assay as described in section 2.4.1. Experiments were duplicated and repeated at least twice. Results were presented as surviving bacterial population in Log_10_ CFUml^−1^ units.

### Cytotoxicity assay

To examine if ACP-based reduction of virulence factors could further influence cytotoxicity of *P*. *aeruginosa*, a cytotoxicity assay using the CHO-K1 cell line was conducted. Detached cells, seeded in 96 well microtiter plates at final concentration of 2.5 x 10^4^ cells/ml (100 μl per well), were supplemented with 10 μl of filter-sterilised supernatant of either ACP treated or untreated (0 h and 24 h controls) *P*. *aeruginosa* cell suspension (filter pore size 0.2 μm). Uninoculated and ACP treated TSB medium were considered as negative controls. Cell growth/adhesion was assessed after incubation at 37°C and 5% CO_2_ in air for 3 days using crystal violet assay (CV). Briefly, after incubation, supernatants were removed and adherent cell layers were fixed with 40 μl 70% methanol for 1 min. After fixation, cells were stained with 50 μl of 0.2% CV solution for 10 min and then extensively washed with water. Adherent CV was dissolved in 10% acetic acid (100 μl per well) and the absorbance was measured at 600 nm on a microplate reader. Results are represented as a percentage of adherent cells determined by the absorbance of the CV, where cell culture supplemented with uninoculated and untreated TSB medium was set as a 100%. Experiments were performed in triplicate and replicated at least three times.

### Statistical analysis

Statistical analysis was performed using SPSS 19.0 (SPSS Inc., Chicago, USA). The surviving populations of challenge bacteria and reduction of pyocyanin and elastolytic activity in *P*. *aeruginosa* following ACP treatment were subjected to analysis of variance (ANOVA). Means were compared according to the method of Fisher’s Least Significant Difference-LSD at the 0.05 level. Absorbance values of CHO-K1 cells obtained from the exposure to untreated TSB, *P*. *aeruginosa* 0 h and 24 h controls and ACP treated for 60, 120 and 300 s of corresponding groups, i.e. the type of media (*P*. *aeruginosa* and TSB) and type of treatment (direct and indirect) were compared according to the method of LSD at the 0.05 level.

## Results

### Effect of ACP on bacterial biofilms

#### Colony count assay

**Fig 2:**
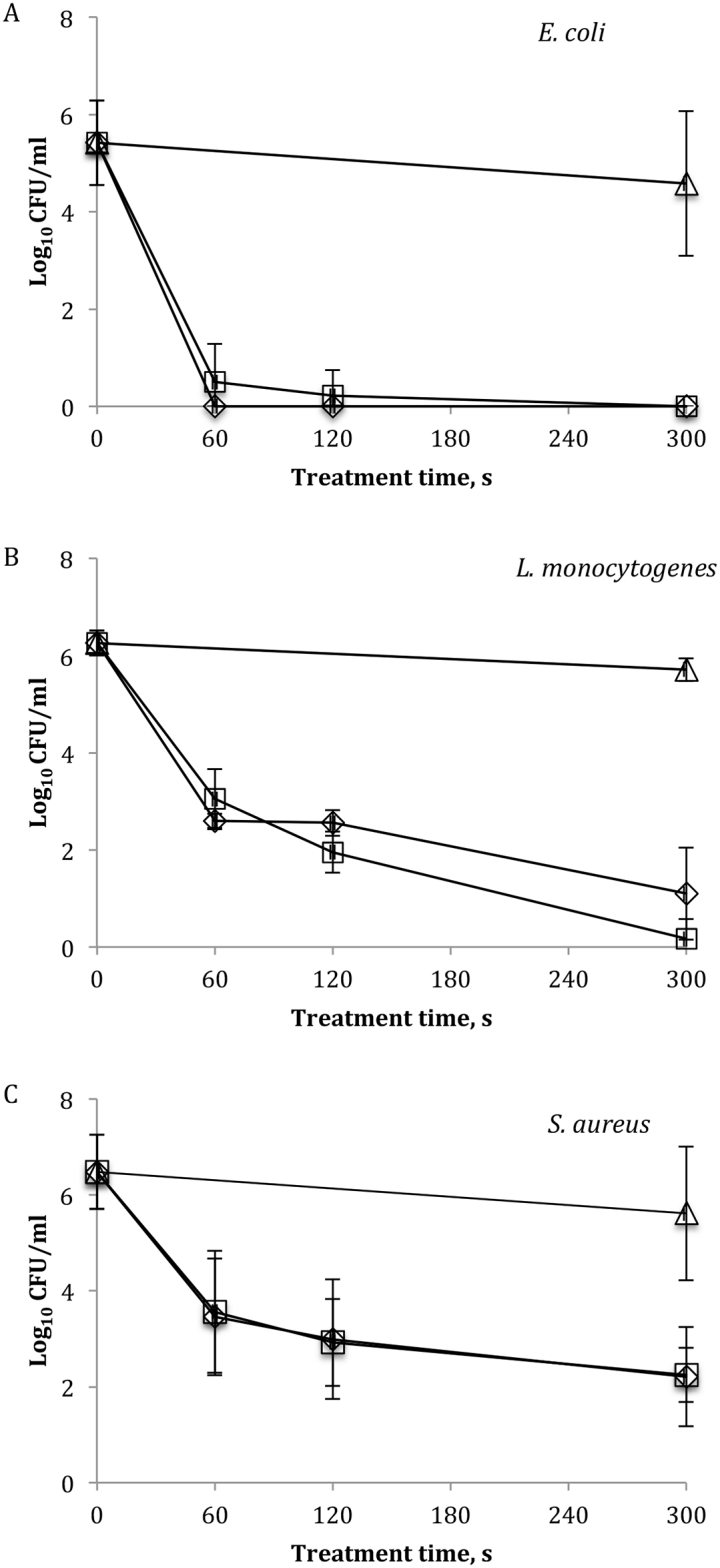
Surviving populations of bacterial biofilms after ACP treatment assessed by colony count assay. (A) *E*. *coli*, (B) *L*. *monocytogenes* and (C) *S*. *aureus*: (Δ) untreated 24 h control, (◊) after direct and (□) indirect ACP treatment. Vertical bars represent standard deviation. Limit of detection 1.0 log_10_ CFU/ml. For each microorganism, all experiments were conducted in triplicate using independently grown cultures and replicated at least twice.

Surviving populations of *E*. *coli*, *L*. *monocytogenes*, *S*. *aureus* 48 h biofilms as a function of treatment time and exposure type are presented in Fig 2. The average initial population of *E*. *coli* recovered from untreated control biofilms was 5.4 ±0.87 log_10_ CFU/ml (Fig 2A). On average, population densities were reduced below the detection limit of 1.0 log_10_ utilising either direct or indirect type of exposure and for all treatment times applied.

*L*. *monocytogenes* biofilm populations were reduced (p ≤ 0.05) from the mean value of 6.3 ±0.25 log_10_ CFU/ml before treatment (0 h control) to an average of 2.8 log_10_ CFU/ml after 60 s of direct/indirect treatment (Fig 2B). Extending treatment time to 120 s using direct exposure did not enhance antimicrobial effect, however, 120 s of indirect treatment reduced cell numbers to an average of 1.9 ±0.42 log_10_ CFU/ml (p ≤ 0.05). Extending treatment to 300 s using direct exposure did yield significant reductions of *L*. *monocytogenes* cells in biofilms to an average of 1.1 ±0.94 log_10_ CFU/ml, and *L*. *monocytogenes* was undetectable when exposed to indirect treatment for 300 s.

The inactivation effect of ACP treatment against *S*. *aureus* biofilms is shown in Fig 2C, where similar reductions were observed with either direct or indirect ACP exposure. However, there was no significant difference between the population density after 60 s of treatment and the 24 h controls. Increasing treatment time did reduce populations further, where the population densities surviving 120 s and 300 s of treatment significantly differed from the 24 h storage control biofilm (p ≤ 0.05).

#### XTT assay

**Fig 3:**
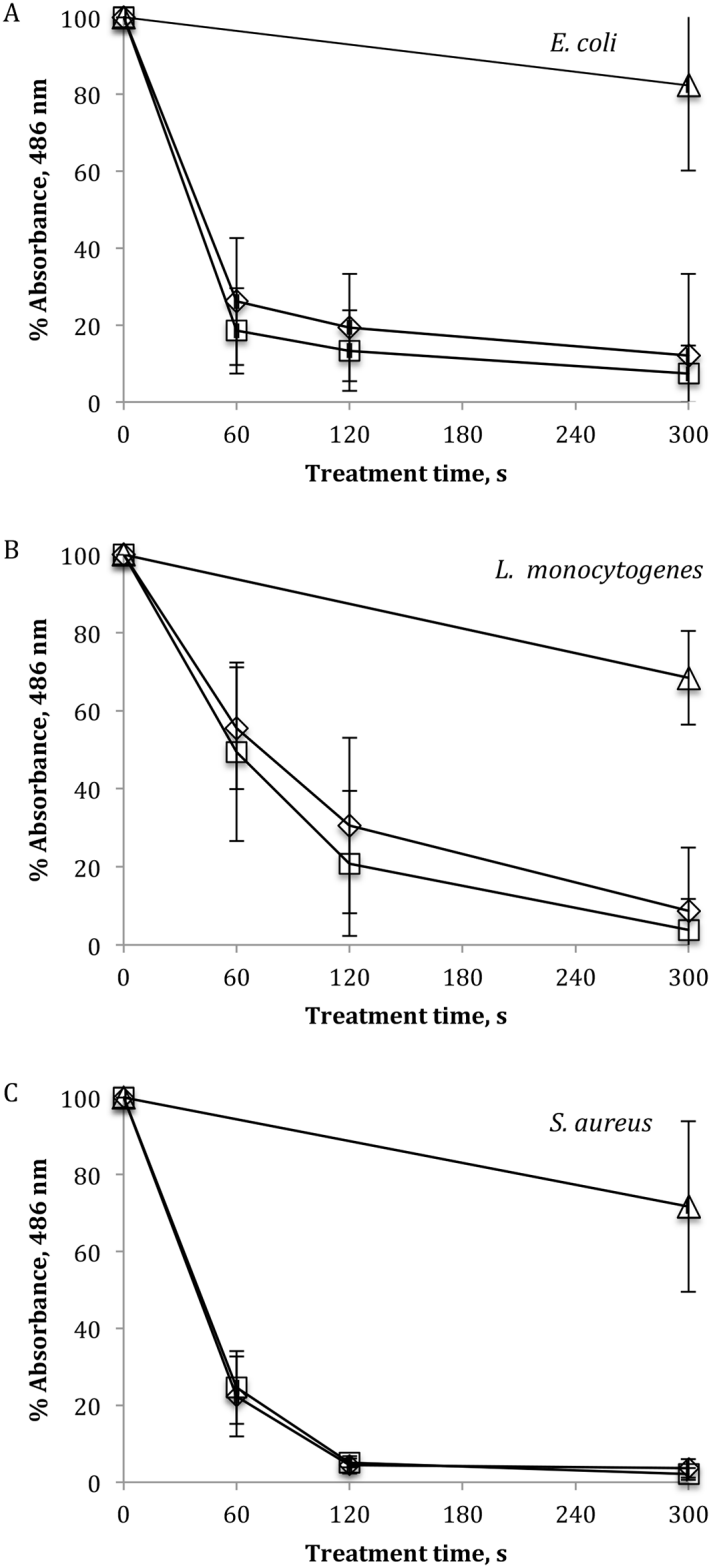
Percentage surviving populations of bacterial biofilms after ACP treatment assessed by XTT assay. (A) *E*. *coli*, (B) *L*. *monocytogenes* and (C) *S*. *aureus*: (Δ) untreated 24 h control, (◊) after direct and (□) indirect ACP treatment. Vertical bars represent standard deviation. For each microorganism, all experiments were conducted in triplicate using independently grown cultures and replicated at least twice.

Effects on viability and metabolic activity of *E*. *coli*, *L*. *monocytogenes*, *S*. *aureus* 48 h biofilms after direct or indirect ACP treatment and 24 h of post treatment storage time are presented in Fig 3. For the three test microorganisms, there was no significant difference found between the effects of direct and indirect ACP exposure. Thus, direct/indirect ACP treatment for 60 s reduced *E*. *coli* cells metabolic activity by an average of 77.7% (Fig 3A). Further increasing treatment time to 300 s yielded a maximum average reduction of 90.0%.

Fig 3B represents the effects of ACP treatment on the metabolic activity of *L*. *monocytogenes* cells in biofilms. XTT absorbance values indicate a correlation between ACP inactivation efficacy with increased treatment time. Direct treatment for 60, 120 and 300 s significantly reduced metabolic activity of *L*. *monocytogenes* cells to an average of 55.5 ±15.7%, 30.6 ± 22.5% and 8.7 ±16.2%, respectively, compared to the untreated 0 h control (p ≤ 0.05). In the case of indirect treatment, a similar trend was observed, where the percentage of metabolically active cells significantly decreased to an average of 49.5 ±22.9%, 20.8 ±18.5% and 3.8 ±7.9% after 60, 120 and 300 s of indirect treatment, respectively, with reference to the 0 h control.

A rapid decline in cell metabolic activity following both direct and indirect ACP treatment was observed for *S*. *aureus* biofilms (Fig 3C). The average percentage of cells surviving 60 s of treatment was 23.5%, which decreased to almost nil as treatment time increased. There was no statistical difference found between percentage absorbance values obtained from 0 h and 24 h controls in the case of the three microorganisms examined.

#### Scanning electron microscopy (SEM)

**Fig 4:**
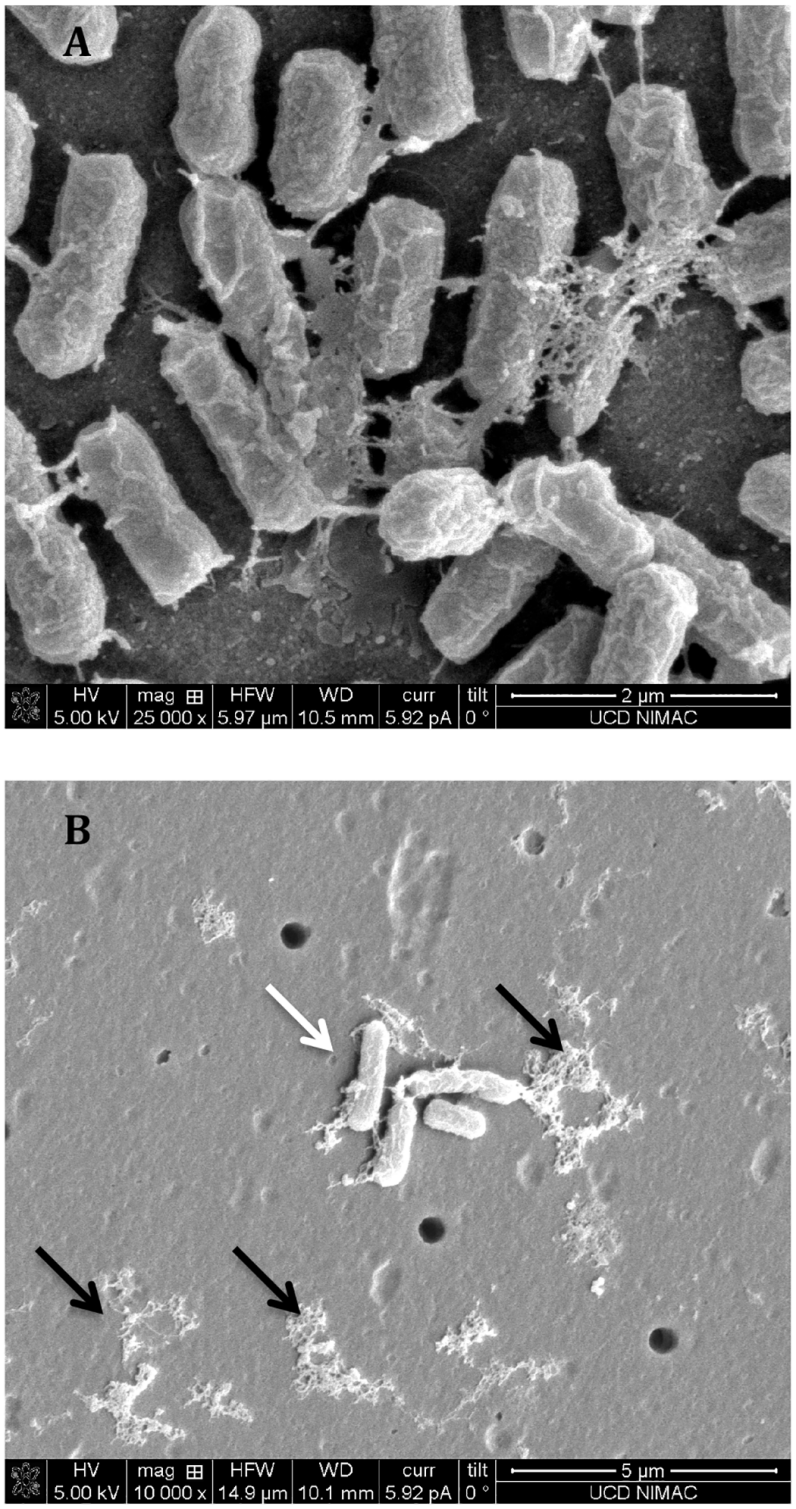
Scanning electron microscopy images of *E*. *coli* 48 h biofilms. (A) untreated 0 h control (magnification 25 000X) and (B) after 300 s of direct ACP treatment (magnification 10 000X). Black arrows indicate cell debris; white arrows indicate intact cells.

SEM analysis was utilised in order to visualise the effect of ACP treatment on bacteria in the form of a complex biofilm structure. Fig 4 represents SEM images obtained for 48 h *E*. *coli* biofilms, showing either untreated 0 h control or biofilm treated with ACP for 300 s using direct exposure. Images of the *E*. *coli* control sample confirmed the presence of healthy cells and biofilm matrix formation (Fig 4A), whereas after ACP treatment, a large proportion of bacterial cells was disintegrated and cell debris fragments were found on the surface of PET membrane (black arrows) (Fig 4B). However, in several areas of the ACP treated membrane, the bacterial cells remained intact (white arrow).

### Effect of ACP on *P*. *aeruginosa* QS-controlled virulence factors

**Fig 5:**
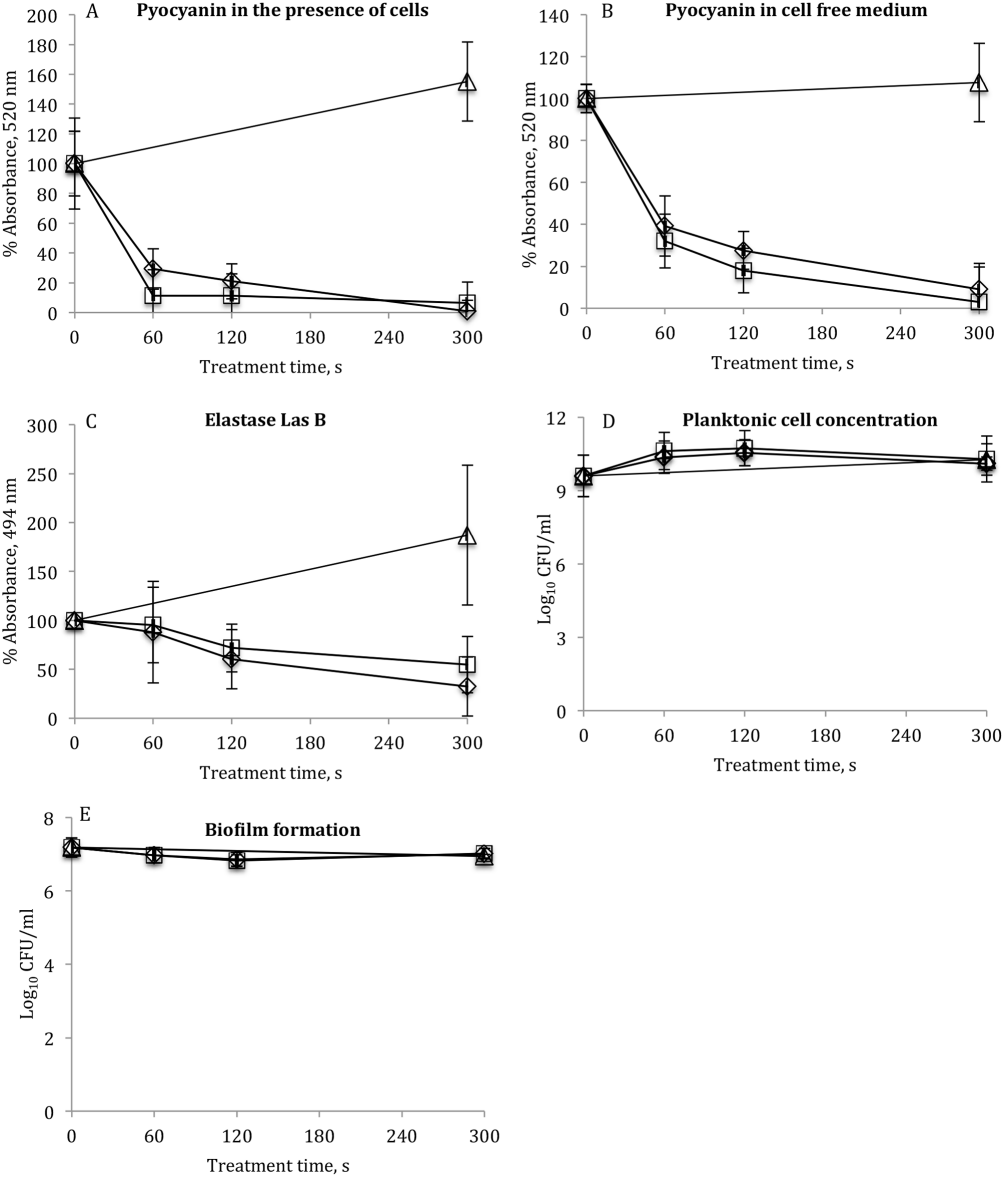
ACP inhibition effects on *P*. *aeruginosa* virulence factors. (A) pyocyanin in the presence of cells, (B) pyocyanin in the cell free medium (experiments repeated at least three times), (C) elastase Las B (experiments repeated at least five times), (D) planktonic cell concentration (experiments repeated at least six times) and (E) biofilm formation (experiments were performed in duplicate and replicated at least twice): (Δ) untreated 24 h controls, (◊) after direct and (□) indirect ACP treatment. Vertical bars represent standard deviation.

The effects of ACP on *P*. *aeruginosa* QS-controlled virulence factors were investigated utilising bacterial cell suspension (Fig 5). In a parallel study *P*. *aeruginosa* cell free supernatant was used to examine any possible effect of cells present during the treatment in the TSB media on the levels of pyocyanin. In general, both direct and indirect ACP treatment resulted in similar reduction of both pyocyanin and elastase levels. The levels of pyocyanin retained after ACP treatment and 24 h of post treatment storage are shown on Fig 5A. Short treatment for 60 s by either direct or indirect ACP significantly reduced concentrations of pyocyanin by 70.7 ±9.68% and 88.5 ±11.34%, respectively (p ≤ 0.05), compared to 0 h control. The levels of pyocyanin in the cell free culture supernatant were reduced by an average of 60.8 ±14.34% and 68.0 ±12.77% after 60 s of direct and indirect treatment (Fig 5B), respectively, whereas 300 s of treatment almost completely inactivated pyocyanin in both the cell suspension and the cell free supernatant (Fig 5A and 5B). In contrast, slower inhibition rates of elastolytic activity were recorded (Fig 5C). According to the absorbance values measured at 495 nm, only direct treatment for 300 s yielded a significant reduction in the elastolytic activity of elastase by comparison with the 0 h control (p ≤ 0.05). However, elastase levels after 300 s of either direct or indirect treatments were lower than the corresponding 24 h untreated controls (p ≤ 0.05). In order to ensure that the reduction of *P*. *aeruginosa* virulence factors was not due to the bactericidal action of ACP, the numbers of planktonic cells in TSB were estimated using colony count assay. From Fig 5D it can be seen that there were no significant changes in bacterial cell population density noted after any of the treatment times applied. The ACP treatments also did not influence the ability of *P*. *aeruginosa* to form biofilms, as there was no reduction in cell populations of the 24 h biofilms initiated from ACP treated cell suspensions (Fig 5E).

### Effect of ACP on cytotoxicity of *P*. *aeruginosa*

**Fig 6:**
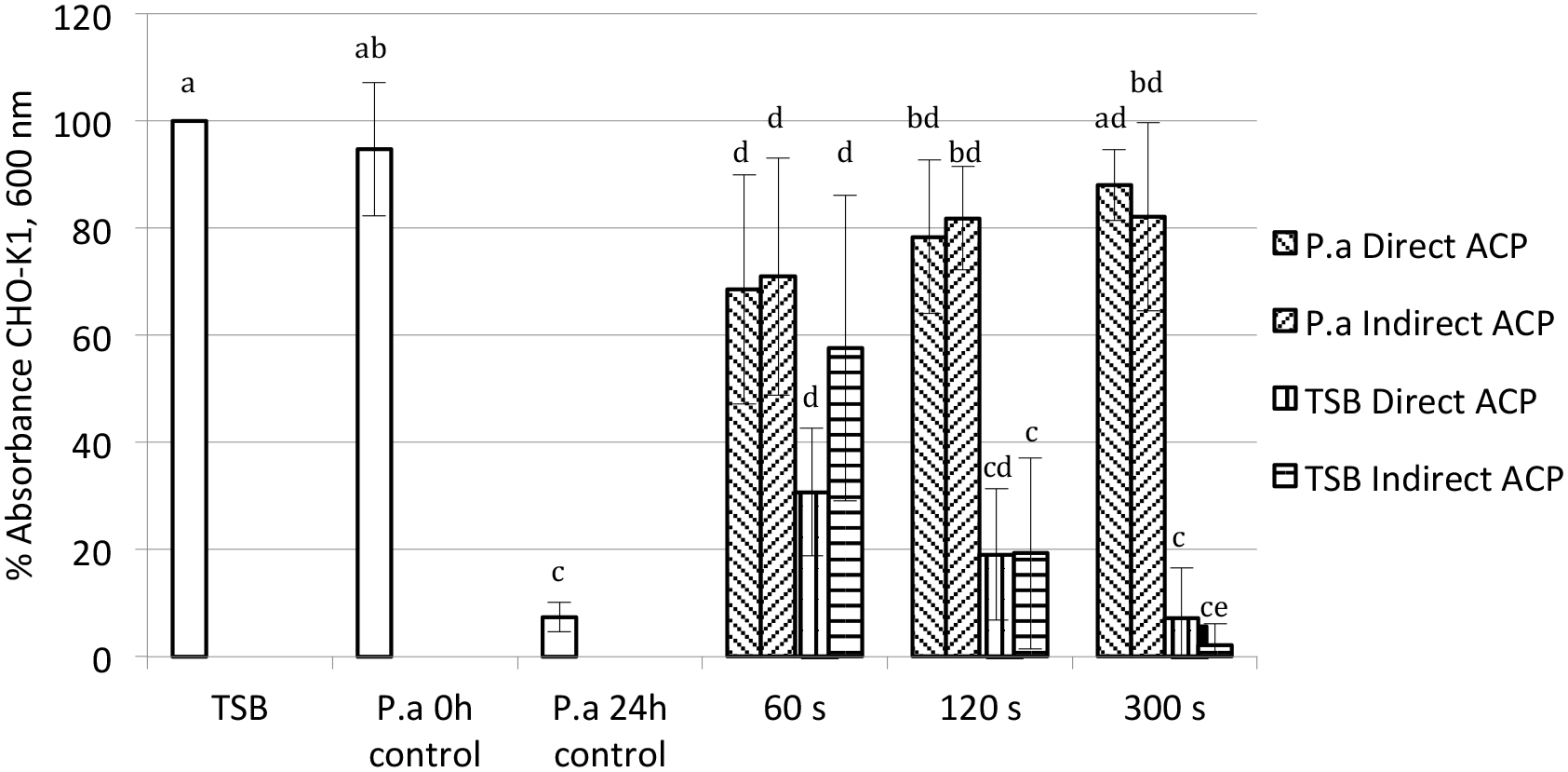
The effect of either untreated (0 h, 24 h controls) or ACP treated *P*. *aeruginosa* (P.a) and ACP treated TSB medium on growth/adherence of CHO-K1 cells. Different letters indicate a significant difference in % absorbance levels. Vertical bars represent standard deviation. Experiments were performed in triplicate and replicated at least three times.

The effects of *P*. *aeruginosa* treated with either direct or indirect ACP and stored for 24 h or untreated and stored for 0 h and 24 h (0 h and 24 h control) on growth/adherence of CHO-K1 cells are presented on Fig 6. Uninoculated TSB subjected to the same treatment were used as controls and untreated TSB was set as 100% cell growth. Significant reduction by an average of 92.6 ±2.70% in cell growth resulted from the exposure of CHO-K1 to untreated *P*. *aeruginosa* 24 h control as compared to the untreated TSB (p ≤ 0.05). Regardless of the type of treatment, exposure of CHO-K1 to supernatant of *P*. *aeruginosa* treated with ACP for 60, 120 and 300 s increased the growth of cells by an average of 62.4, 72.7 and 77.7%, respectively, by comparison with *P*. *aeruginosa* 24 h untreated controls (p ≤ 0.05).

While there was no significant difference recorded between the growth of CHO-K1 cells as a result of different treatment times applied against *P*. *aeruginosa* cell suspensions, a treatment time dependent decrease in cell adherence was observed when CHO-K1 were exposed to uninoculated and ACP treated TSB medium, where increasing treatment time resulted in reduction of cell growth by an average of 55.9, 80.8 and 95.4%, respectively. Absorbance values of cultures exposed to uninoculated TSB treated with ACP for 120 and 300 s were significantly lower than the values obtained from cells exposed to ACP treated *P*. *aeruginosa* supernatants (p ≤ 0.05).

## Discussion

Microbial biofilms form on a wide variety of surfaces, including living tissues, indwelling medical devices, industrial or potable water systems, food and food-contact surfaces, thereby establishing reservoirs for continuous contamination [4, 30]. In response to an increasing tolerance of microorganisms to broad-spectrum antibiotics, and resistance of biofilms in particular, research has focussed on achieving an efficient inactivation and removal of bacterial biofilms. Among the various methods, ACP technology demonstrated remarkable effectiveness against a range of microorganisms, including antibiotic-resistant biofilm forming strains [31].

In the current study, a dielectric barrier discharge (DBD) configuration was used to generate high voltage ACP, and investigated against a range of microbial biofilms commonly implicated in foodborne and healthcare associated human infections, *E*. *coli*, *L*. *monocytogenes* and *S*. *aureus*. Either direct or indirect atmospheric air ACP treatment was highly effective against challenge 48 h biofilms with no significant difference in inactivation efficacy observed between the modes of ACP exposure. The 24 h of post treatment storage time used in conjunction with in-package ACP treatment allowed the retention of plasma generated reactive species inside the pack over time, facilitating bactericidal action of both types of exposure. It is likely that the reductions obtained after indirect treatment would not be possible to achieve without species retention, as the action of treatment is mainly based on the reaction of long lived and recombined species rather than on the combined action of charged particles positive and negative ions, electrons, free radicals, excited and non-excited molecules and atoms associated with direct treatment. The efficiency of ACP treatment was found to be bacterial type dependant. Biofilms by their nature are complex biomaterials, the composition of which will vary between the type and strain of bacteria [32]. According to colony counts, treatment for 60 s reduced populations of Gram-negative *E*. *coli* biofilms to undetectable levels, whereas this treatment time was less effective for Gram-positive *L*. *monocytogenes* and *S*. *aureus* biofilms (Fig 2). Although inactivation of *L*. *monocytogenes* biofilms was slower by comparison with the rapid reduction of *E*. *coli*, an extended direct treatment for 300 s reduced *L*. *monocytogenes* cells by > 5.0 log_10_ CFU/ml and cells recovered after indirect ACP were below detection limits. Large proportions of *S*. *aureus* biofilm populations were inactivated after 60 s of treatment (~3.0 log10 CFU/ml) with only a small further reduction achieved after treatment time of 300 s, indicating a stronger resistance of this strain. It has been reported that Gram-positive bacteria are more resistant to ACP treatments than Gram-negative bacteria [33–36]. The membrane structure of the Gram-positive bacteria may present a barrier to the diffusion of ACP reactive species through the bacterial cell wall, thus impacting ACP antimicrobial efficacy. The different modes of ACP action against Gram-positive bacteria and Gram-negative have been also reported. For example, cell membranes of *L*. *innocua* remained intact during ACP treatment and ACP generated species reacted with cellular components [37], whereas both the cell membrane and cellular components of *E*. *coli* were affected. In contrast, there was no difference in inactivation efficacy of ACP between Gram-negative and Gram-positive bacteria [38], which suggested that there is no selectivity in the action of ACP generated species based on the bacterial cell wall structure. Moreover, the thickness, composition and quantity of the EPS may vary depending on the type of microorganisms [32], which could consequently impact ACP inactivation efficacy. These questions remain regarding the ACP mechanism of action on bacterial biofilms. In the current work, in addition to the difference between Gram-positive and Gram-negative bacteria, different ACP inactivation efficacy was noted for the reductions of biofilms of the two Gram-positive bacteria, *L*. *monocytogenes* and *S*. *aureus*, where *S*. *aureus* exhibited higher resistance to treatment than *L*. *monocytogenes*. These observations could be related to the morphology of bacterial cells, where spherical-shaped *S*. *aureus* (cocci) may be more resistant to ACP than rod-shaped *L*. *monocytogenes*. Similarly, the inactivation efficacy of ultrasound can be influenced by the shape of bacterial cells with more resistant spherical cells than rod-shaped cells [39]. However, the effects of ACP treatment on different bacteria possessing different cell shape remain unclear and warrant further investigation.

Colony count assay estimates the number of cells that survive treatment and are able to grow on TSA agar but does not account for cells that might be metabolically active but unable to grow. XTT assay was conducted in order to detect the retention of metabolic activity of bacterial cells exposed to ACP treatment (Fig 3). In general a good correlation between results obtained from XTT and colony counts was found in the case of Gram-positive *L*. *monocytogenes* and *S*. *aureus* biofilms. However, corresponding results for Gram-negative *E*. *coli* indicated that although 60 s of treatment was able to reduce bacterial counts to undetectable levels, complete inactivation of metabolic activity could not be achieved even after 300 s. It is known that under one or more environmental stresses bacteria may enter Viable-But-Non-Culturable (VBNC) or so-called dormant state [40], a strategy that bacteria employ to tolerate conditions that are detrimental to growth [41]. The results obtained from XTT assay indicated that bacteria were in a VBNC state due to oxidative stress encountered from chemically reactive species generated by ACP. These results were supported by SEM analysis conducted to examine changes of *E*. *coli* biofilm morphology after direct ACP treatment for 300 s (Fig 4). The images of treated biofilms confirmed the bactericidal action of ACP showing significant changes in biofilms, with the structures altered from healthy cells interconnected by self-produced EPS matrices, to irregularly shaped cell fragments. However, although most of the cells were disintegrated, intact cells were found after exposure to ACP, suggesting that treatment could not inactivate bacteria completely as determined by colony count assay. Surviving cells present on ACP treated membrane indicate retention of cell metabolic activity, i.e. presence in their VBNC sate. Recently it has been demonstrated that, although non-culturable, *P*. *aeruginosa* retained its virulence after relatively short time of ACP exposure resulting in lettuce tissue damage [42]. Microbial pathogens in such a state can retain virulence and also pose further challenge as stressed cells may be of a higher virulence potential than bacteria exposed to favourable growth conditions [43].

*P*. *aeruginosa* is known for its diverse pathogenicity and is an ubiquitous, metabolically versatile opportunistic pathogen that causes a wide variety of acute and persistent infections in humans and is resistant to broad spectrum antibiotic treatments [44]. The resistance of *P*. *aeruginosa* to antimicrobials is generally attributed to the reduced cell wall permeability, active multidrug efflux systems, biofilm formation, plasmid acquisition and mutation due to the stress encountered from continuous antibiotic use [13, 45]. Inhibition of QS-controlled virulence factors is a novel approach with a potential to decrease or substitute the use of traditional antibiotics. Recent investigations of the effects of natural and chemically synthesised compounds have demonstrated that reduction of virulence factor production and prevention of biofilm formation with aims to control microbial infection is possible through inhibition of one or more components of the QS system [46–50]. Unfortunately, many known QS inhibition compounds are themselves cytotoxic at practically useful concentrations, thus limiting their topical and internal applications in mammalian systems. The potential of ACP treatment to interfere with virulence of *P*. *aeruginosa* was recently demonstrated by Vandervoort and Brelles-Marino [42], which showed attenuated bacterial virulence after longer treatment duration as tested by the lettuce assay. Our previous studies demonstrated that in-package ACP treatment was highly effective against established *P*. *aeruginosa* 48 h biofilms by disintegrating both bacterial cells and the biofilm matrix [51], which prompted further investigations whether this technology is capable of interfering with *P*. *aeruginosa* QS-controlled virulence factors, such as pyocyanin and extracellular elastase (Las B) and as a consequence affect biofilm formation (Fig 5A, 5B, 5C and 5E). Thus, in the current study, in order to rule out any underlying bactericidal effects of ACP, the changes in the planktonic cell population density were monitored by conducting colony count assay following treatment (Fig 5D). Short treatment time of 60 s in conjunction with either type of exposure significantly reduced *P*. *aeruginosa rhl*-regulated pyocyanin levels, with effects retained during 24 h of post treatment storage time at room temperature. Atmospheric air ACP is a significant source of multiple reactive oxygen species, including ozone, atomic oxygen, singlet oxygen, superoxide, peroxide, hydroxyl radicals and excited nitrogen species [52–53]. Therefore, pyocyanin could undergo oxidation by ROS and RNS generated during ACP treatment. Similar oxidative effects were observed in [54–56], which reported irreversible oxidation of pyocyanin by hydrogen peroxide, singlet oxygen and nitrite, significantly reducing its cytotoxic/proinflamatory activity. Another virulence factor studied; elastase (Las B), is mainly under the control of the *las* system and plays an important role in biofilm development [17, 46, 57]. While concentrations of pyocyanin were significantly reduced after short treatment times, reduction of extracellular elastase was notable only after extended treatment time and when compared with the 24 h control samples. Importantly, the reductions of the studied virulence factors were associated with no reduction in the planktonic cell concentration. Reduced bactericidal effects of ACP treatment against planktonic cells could be due to protective effect of TSB where media components are likely to scavenge many of the plasma reactive species generated during the treatment. While it was hypothesized that the disruption of QS systems could further lead to the reduction in *P*. *aeruginosa* biofilm formation, no reduction in biofilm formation capacity was achieved even after extended treatment for 300 s. The QS pathways regulating biofilm formation are complex and despite the demonstrated activity of ACP against two of *P*. *aeruginosa*’s virulence factors, the effect on QS system and other biofilm formation mechanisms will need to be further elucidated.

The toxic effects of *P*. *aeruginosa* QS-controlled virulence factors on mammalian cells, including elastase and pyocyanin, have been widely reported: a concentration dependent cytotoxic effect of pyocyanin on different eukaryotic systems was reported in [58] and [59], whereas *P*. *aeruginosa las B* mutant strain almost totally abrogated epithelial cell cytotoxicity [60]. In this study, ACP-mediated reduction of *P*. *aeruginosa* virulence factors was associated with significant reduction of cytotoxic effects of bacterial supernatant on CHO-K1 cells regardless of type and duration of treatment. These results indicate the high potential of ACP treatment to attenuate bacterial virulence within relatively short treatment times (60 s), which could prove valuable for future in vivo ACP applications targeting internal or indeed surface infections or as a mode of low toxicity pre-emptive infection control. However, a pronounced decrease in viability of CHO-K1 was observed when cells were exposed to the corresponding uninoculated and ACP treated TSB medium. The ACP treatment time dependent reduction of cell viability could be due to the action of ROS and RNS accumulated and retained in the medium during the treatment, with little interference presented by the relatively simple constituents. Wende *et al*. [61], exposed human keratinocytes, HaCaT cells, to ACP treated media and found a treatment time dependent increase in accumulation of intracellular ROS. Moreover, intracellular ROS levels strongly depended on the cell culture medium, where small organic molecules such as sugars, amino acids, vitamins and buffer systems could interfere with ROS stability/propagation within the liquid, modifying any plasma effects. In contrast, Haertel *et al*. [62], exposing HaCaT cells either to air ACP or to the ACP treated media found that the levels of intracellular ROS induced were neither dependent on the duration of the plasma treatment nor the treatment type. Furthermore, exposure either to ACP treatment or ACP treated media resulted in a comparable reduction in the levels of adherent HaCaT cells. Together with our observations these results indicate the additional effects of ACP treatment on non-target cells or systems, which need to be considered during development of anti-microbial, anti-QS or anti-virulence therapies, but does not exclude the potential use of ACP treated fluids for human application. It is also important that exposure to ACP treated TSB media significantly reduced cell growth by comparison with the effects of *P*. *aeruginosa* supernatants, which could be due to the ROS scavenging properties of multiple components secreted into a medium by this pathogen, including alginate and pigments, such as pyocyanin, pyoverdine and pyorubin [63]. In recent years ACP has been under intense investigation for potential applications in wound healing, blood coagulation and skin regeneration, and apoptosis of cancer cells [64–70]. Apart from ACP-mediated induction of cell proliferation, lethal effects on mammalian cells have also been reported [71–72]. Therefore further investigations should focus on the effects of ACP treatment where the balance between ACP bactericidal action and effects on mammalian cell viability will be the key factor determining the system’s potential in chronic and acute therapeutic applications.

The efficacy observed against some monoculture biofilms but not against others highlights the need to examine multispecies biofilm formations where relevant. Although monoculture biofilms are often encountered in clinical settings, this is not the case for environmental occurrences and on foods. It has been known that formation of biofilms is influenced by environmental conditions and characteristics of substrates to which bacteria attach. In this study the model system, a 96 well microtiter plate, and relatively short time frame was utilised for biofilm development, which cannot holistically describe the broad range of materials implicated in biofilm formation within clinical environments and industrial settings. Therefore, further studies on the efficacy of ACP against multispecies biofilms developed on a wider range of relevant materials are warranted. It is considered that QS-regulated virulence factors, such as pyocyanin and elastase, play a crucial role in clinical pathogenicity of *P*. *aeruginosa* [73] and inhibition of these virulence factors is a key element in the future research for the replacement of current antibiotics. To date, this is the first report focusing on anti-virulence activity of ACP, which clearly demonstrated that, although there was no antibiofilm effect observed against *P*. *aeruginosa* at the parameters examined, the treatment exhibited high potential against QS-regulated virulence factors and these observations may further serve as a means for exploration of new ACP-based strategies for tackling infections caused by this microorganism.

## Conclusion

The results of this work clearly demonstrated that ACP could be a potential strategy for inactivation of the established *E*. *coli*, *L*. *monocytogenes* and *S*. *aureus* 48 h biofilms. However, the type of bacteria in conjunction with its biofilm composition might have an effect on ACP inactivation efficacy. Furthermore, despite the ability of ACP treatment to penetrate and destroy bacterial cells within complex biofilm structures, the bacterial metabolic state and cell morphology could be important factors in determining the inactivation efficacy. Importantly, significant reductions of *P*. *aeruginosa* QS-regulated virulence factors, such as pyocyanin and elastase (Las B), were achieved, suggesting that ACP technology may play an important role in attenuation of virulence of pathogenic bacteria. However, even after 300 s treatment, biofilm formation capacity was largely unaffected, suggesting that the inactivation of virulence factors by ACP could also be independent of QS mechanisms. Further optimization of ACP treatment parameters for successful prevention and inactivation of established and developing biofilms should focus on the mechanisms involved in the ACP-mediated reduction of virulence factors and complete inactivation of VBNC state to reduce the risks associated with cross- or re-contamination during or after treatment.

## Supporting Information

S1 Table Dataset for experimental results. (XLSX)
